# A Multifactorial Approach to Sleep and Its Association with Health-Related Quality of Life in a Multiethnic Asian Working Population: A Cross-Sectional Analysis

**DOI:** 10.3390/ijerph16214147

**Published:** 2019-10-28

**Authors:** Gerard Dunleavy, André Comiran Tonon, Ai Ping Chua, Yichi Zhang, Kei Long Cheung, Thuan-Quoc Thach, Yuri Rykov, Chee-Kiong Soh, Georgios Christopoulos, Hein de Vries, Josip Car

**Affiliations:** 1 Centre for Population Health Sciences, Lee Kong Chian School of Medicine, Nanyang Technological University Singapore, 11 Mandalay Road, Singapore 308232, Singapore; 2 Laboratório de Cronobiologia e Sono, Porto Alegre Clínicas Hospital (HCPA), Porto Alegre 90035-007, Brazil; 3 Postgraduate Program in Psychiatry and Behavioral Sciences, Federal University of Rio Grande Do Sul (UFRGS), Porto Alegre 90040-060, Brazil; 4 Department of Medicine, Jurong Health Campus, National University Health System, 1 Jurong East Street 21, Singapore 609606, Singapore; 5 Department of Clinical Sciences, College of Health and Life Sciences, Brunel University London, London UB8 3PH, UK; 6 School of Civil and Environmental Engineering, College of Engineering, Nanyang Technological University Singapore, 50 Nanyang Avenue, Singapore 639798, Singapore; 7 Division of Leadership, Management and Organisation, Nanyang Business School, College of Business, Nanyang Technological University Singapore, 50 Nanyang Avenue, Singapore 639798, Singapore; 8 Department of Health Promotion, CAPHRI Care and Public Health Research Institute, Maastricht University, Maastricht POB 616 6022 MD, The Netherlands; 9 Department of Primary Care and Public Health, School of Public Health, Imperial College London, London SW7 2AZ, UK

**Keywords:** sleep quality, quality of life, workplace health

## Abstract

This study aims to explore if objectively and subjectively measured sleep parameters are associated with physical and mental health-related quality of life in a multiethnic working population in Singapore. We performed a cross-sectional analysis with data from 329 full-time employees enrolled in a workplace cohort study in Singapore. The Short-Form 36v2 (SF-36v2) survey was used to assess health-related quality of life, in terms of physical and mental health. Subjective and objective sleep parameters were measured using the Pittsburgh Sleep Quality Index and wrist actigraphy, respectively. Generalized linear modeling was performed to examine the association between sleep parameters and health-related quality of life. After adjusting for confounders, subjectively measured sleep disturbances were associated with a lower physical health-related quality of life, whereas higher, objectively measured sleep efficiency was associated with greater physical health-related quality of life. Subjectively measured daytime dysfunction was associated with impaired mental health-related quality of life. Using both objective and subjective measurements of sleep, the current study suggests that there is an association between sleep and health-related quality of life. Workplace health-promotion planners in Singapore should consider programmes that educate workers on better sleep hygiene practices in an effort to improve sleep and health-related quality of life.

## 1. Introduction

The sleep-wake cycle is a complex phenomenon that encompasses several physiological and behavioral oscillations [1]. Although no consistent evidence has proven the reasons why humans sleep [2], a growing body of literature shows that low sleep quantity or poor sleep quality is associated with hypertension, heart disease, stroke, diabetes, and even cancer, as well as mood disorders, commonly anxiety and depression [3,4]. Other than physical and mental ailments, symptoms of sleepiness and fatigue from sleep deficiency during wake periods can lead to impaired performance, reduced work productivity, increased workplace absenteeism, and risk of work-related accidents [3]. In a recent study, residents of Singapore, a city-state that is heavily reliant on its human capital, were reported to have the shortest sleep duration of the 20 countries enrolled in the study [5]. Therefore, the negative effects of sleep deprivation and poor sleep quality are of concern in Singapore. The lack of sleep among residents of Singapore is often linked to the well-established culture of working long hours. Full-time workers in Singapore worked an average of 45 h per week in 2019 [6]. In contrast, the average full-time worker in the U.S. and the EU worker spent 35 h and 42 h working per week, respectively [7]. Another challenge to both sleep quality and duration for residents of Singapore is the excessively floodlit society in which they live as the city-state is reported to be the most light-polluted country in the world [8]. Given the prevalence of poor sleep quality, daytime fatigue, and short sleep duration of residents in Singapore [5,9], research is needed to examine how sleep impacts the health-related quality of life of working populations in Singapore.

Quality of life is a multidimensional concept encompassing physical, psychological, and social domains, all of which are shown to be negatively impacted by reduced sleep quantity or poor sleep quality [10]. The 36 Item Short Form Health Survey questionnaire (SF-36) is a generic instrument that has been used to measure, assess, and evaluate the quality of life of different populations in Singapore [11,12,13]. The SF-36 assessment produces two summary measurements of health-related quality of life, namely, a physical component summary score which represents physical health-related quality of life, and a mental component summary score which represents mental health-related quality of life. Considering the multifaceted nature of sleep, there is no consensus on what is the best variable to measure and report for the study of physical and mental health-related quality of life. Common direct subjective measurements of sleep are self-reported sleep quality and satisfaction, average sleep duration, and sleep efficiency (sleep latency and wake after sleep onset). Indirect subjective measurements include self-perceived sleepiness and unintentional napping during awake working periods, as well as work and academic performance. The Pittsburgh Sleep Quality Index (PSQI) which was introduced in 1989 has remained one of the most popular, well-established and validated clinical tools used to collect both direct and indirect measurements of subjective sleep quality [14], albeit its limitations. Direct objective measurements of sleep duration and quality are actigraphy and polysomnogram (PSG), and indirect measurements are mean sleep latency test, mean wakefulness test, and various forms of vigilance performance testings. Actigraphy has emerged as an attractive, practical and cheaper alternative to PSG, which has been extensively validated. Unlike PSG, it allows for prolonged continuous periods of sleep evaluation in the subject’s natural work and sleep environments without being labor-intensive [15,16]. A number of studies have reported on the association between subjectively assessed parameters of sleep and health-related quality of life [17,18,19,20,21,22,23,24,25], while fewer have examined their relationship with objective measurements of sleep [26]. Given the difference in cost, accessibility, and ease of administration, it is understandable that subjective assessments of sleep are often opted for over objective measurements, however, the extant literature has demonstrated inconsistencies between subjectively and objectively measured sleep [27,28,29], and therefore studies that include both methods are warranted. A further gap that exists in the literature is the lack of studies that examine the relationship between sleep and health-related quality of life in working populations, with the majority of studies that investigate this relationship conducted among specific clinical populations [23,30,31,32,33]. To our knowledge, no study has investigated whether sleep is associated with physical and mental health-related quality of life in a working population in Singapore. 

The goal of this study is to explore if sleep parameters are associated with physical and mental health-related quality of life in a multiethnic working population in Singapore. We aim to describe the independent effects of subjective sleep parameters and objective actigraphy-based data, accounting for confounding factors (i.e., sociodemographic characteristics, lifestyle, and occupational factors).

## 2. Materials and Methods

### 2.1. Study Design and Sample

We conducted a cross-sectional analysis of data of 329 adults (21 years and above) who were participating in a workplace cohort study in Singapore. Details of the cohort study design are published elsewhere [34]. Briefly, a cohort of 464 full-time employees was set up in four workplaces in Singapore to study the effects of working underground vs. aboveground on sleep quality and melatonin levels. The four workplaces included companies from the transport industry, cooling plants, and universities. Participants from these workplaces were invited to participate in the study via meetings, workplace posters, and emails. Those willing to participate were then screened for eligibility using the following criteria: aged 21 years and above, should be working for at least four hours per day, not pregnant, and had not traveled overseas across a different time zone within the past six months. 

### 2.2. Measurements

#### 2.2.1. Health-Related Quality of Life

The Short Form 36 (SF-36v2) survey form was used to assess the health-related quality of life (HRQoL) of participants [35]. The SF-36v2 dimensions are divided into the following two categories: physical component summary (PCS) and mental component summary (MCS), which represent physical and mental health-related quality of life, respectively, and will be referred to as such hereafter in this article. Physical health-related quality of life has 21 items and measures the following four domains: physical functioning (10 items, the following items are about activities you might do during a typical day, example question, “Does your health now limit you in these activities? If so, how much? Lifting or carrying groceries?” Yes, limited a lot, yes, limited a little, no, not limited at all); role limitations due to physical health (4 items, example question, “During the past 4 weeks, have you cut down the amount of time you spent on work or other activities as a result of your physical health?” yes or no); bodily pain (2 items, example question, “During the past 4 weeks, how much did pain interfere with your normal work (including both work outside the home and housework)?” not at all, a little bit, moderately, quite a bit, extremely); and general health perception (5 items, example question, “I am as healthy as anybody I know.” definitely true, mostly true, don’t know, mostly false, or definitely false). Mental health-related quality of life contains 14 items and measures the following four domains: social functioning (2 items, example question, “During the past 4 weeks, how much of the time has your physical health or emotional problems interfered with your social activities (like visiting with friends, relatives, etc.)?” not at all, slightly, moderately, severe or very severe); role limitations due to emotional problems (3 items, example question, “During the past 4 weeks, have you accomplished less than you would like as a result of any emotional problems (such as feeling depressed or anxious)?” yes or no); vitality (4 items, example question, “During the past 4 weeks, did you have a lot of energy?” all of the time, most of the time, a good bit of the time, some of the time, a little bit of the time or none of the time); and emotional well-being (5 items, example question, “During the past 4 weeks, have you been a very nervous person?” all of the time, most of the time, a good bit of the time, some of the time, a little bit of the time or none of the time). The scoring of the two summary components was performed using the Health Outcomes Scoring Software 5.1 (QualityMetric Health Outcomes™, Lincoln, UK) based on 2009 U.S. norms with a mean of 50 and a standard deviation of 10. Higher scores indicate a better health-related quality of life.

#### 2.2.2. Sleep Parameters

##### Subjective Measurements

The Pittsburgh Sleep Quality Index (PSQI) measures sleep quality during the past month [14]. It has 19 self-rated items, which are grouped into seven subscales: subjective sleep quality (1 item), sleep latency (2 items), sleep duration (1 item), habitual sleep efficiency (3 items), sleep disturbances (9 items), use of sleeping medications (1 item), and daytime dysfunction (2 items). Subjective sleep quality was assessed with the PSQI item ”During the past month, how would you rate your sleep quality overall?”. The sleep latency subscale examines how long the participant takes to fall asleep, while the sleep duration subscale examines how many hours of actual sleep the participant gets at night. Habitual sleep efficiency was calculated as follows: hours of sleep/(get up time − usual bedtime) × 100. Sleep disturbance was calculated from the PSQI items “wake up in the middle of the night or early morning”, “need to get up to use the bathroom”, “cannot breathe comfortably”, “cough or snore loudly”, “feel too cold”, “feel too hot”, “have bad dreams”, and “have pain”. Daytime dysfunction was calculated from the PSQI items, “During the past month, how often have you taken medicine to help you sleep (prescribed or over the counter)?” and “During the past month, how often you have you had trouble staying awake while driving, eating meals, or engaging in social activity?”. The score for each component ranges from 0 to 3, with higher scores indicating greater difficulty. The sum of scores for these seven components provides a global score (ranges from 0 to 21), with “0” indicating no difficulty and “21” indicating severe difficulties in all areas. Participants with a PSQI global score >5 were categorized as having poor sleep quality, as per the recommended cut-off by the developers of the screening tool and subsequent validation studies of the instrument [14,36]. Standard scoring of the PSQI was applied.

##### Objective Sleep Measurements

Participants also wore an Actiwatch (Actiwatch Spectrum Plus, Phillips Respironics), which contains an accelerometer capable of estimating locomotor activity (e.g., movement, rest and activity periods). Participants were requested to wear the Actiwatch on their non-dominant wrist, 24 h a day, for a period of eight consecutive days. The participants were instructed on how to use the device by trained staff and they were also requested to complete a sleep diary on a daily basis. Each actigraph was evaluated by a scorer who used a standardized approach to set rest intervals (periods when the subject was trying to sleep) based on 3 inputs: event markers, sleep diary, white light intensity and activity. Actigraphy data were aggregated in 30 s epochs and scored manually as sleep or wake using a procedure published by the NSRR and based on (a) an event marker on the ActiWatch device, (b) the sleep diary, and (c) activity and ambient light data from the ActiWatch device [37]. Participants were instructed to press the event marker (a button on the side of the ActiWatch device) when going to sleep for the evening and when awoken in the morning. The actigraphy variables being reported herein included: (1) total sleep time (hours), which reflects the sum of those epochs between “sleep start” and “sleep end” that were designated as “sleeping”; (2) sleep efficiency (percent %), which is an index of the amount of time in bed actually spent sleeping, is determined by dividing total-sleep-time by the time-in-bed, multiplied by 100; and (3) wake after sleep onset (WASO), which refers to periods (minutes) of wakefulness occurring after sleep onset. 

#### 2.2.3. Confounding Factors 

##### Health and Lifestyle Factors

Smoking status of participants was classified as a non-smoker, ex-smoker, and current smoker. Alcohol drinking status was defined as non-drinker, drinks less than once a month, and drinks once or more than once a month. Physical activity was measured using the Global Physical Activity Questionnaire, which measures activity levels in three domains, namely, work, travel, and leisure. A metabolic equivalent (MET) value of four was assigned for moderate physical activities and a MET value of eight for vigorous physical activities. The duration (in minutes) of an activity performed in each of the three domains was multiplied by its MET value, and these were summed to obtain the total MET-min/week. Individuals with a total MET-min/week < 600, 600–2999, and ≥3000 were considered to be less active, moderately active, and highly active, respectively. Body mass index (BMI) was calculated as weight in kilograms (kg) divided by height in meters (m) squared. Self-reported chronic conditions were assessed using questions on the history of various chronic medical conditions including diabetes, heart disease, stroke, high cholesterol, hypertension, chronic kidney disease, peripheral vascular disease, asthma, allergy, and mental disorders. Stress at home, stress at work, and financial stress were each assessed with single-item questions [38].

##### Occupational Factors 

Participants were asked about their work location (underground or aboveground); shift work (yes or no); job type (office, control room, and workshop); number of work hours per week; and number of years working for the present company.

### 2.3. Statistical Analysis 

Baseline characteristics of participants and the distribution of objective and subjective sleep parameters are summarized using mean (SD: standard deviation) or median (IQR: inter-quartile range) for continuous variables and using frequency and percentage for categorical variables. This study has two dependent variables, physical and mental health-related quality of life. To assess whether various sleep parameters were independently associated with health-related quality of life, we fitted four generalized linear models (GLM) with a Gaussian distribution family using a robust standard error in the following hierarchical fashion, adjusting for sociodemographic characteristics, health and lifestyle factors, and occupational factors. For the first model examining physical health-related quality of life, confounding factors were added in a hierarchical fashion and then the objective sleep parameters were subsequently addedto the final model. For the second model examining physical health-related quality of life, confounding factors were similarly added and then subjective sleep subscales were subsequently included. The same modelling approach was applied to examine mental health-related quality of life with separate analyses performed with subjective and objective sleep parameters. Results for the GLM analysis were expressed as adjusted regression coefficients with 95% confidence intervals using robust standard errors. A negative coefficient reflects a decrease in physical and mental health-related quality of life and a positive coefficient indicates an increase. A two-tailed *p*-value < 0.05 was considered to be statistically significant. Pearson’s correlations were performed to determine the strength of the relationship between subjective and objective sleep parameters. Objective and subjectively measured sleep duration were compared, as were objectively and subjectively measured sleep efficiency. Objectively measured wake after sleep onset was considered a proxy for sleep disturbances, thus the correlation of these variables was examined. All statistical analyses were conducted using Stata 15.0 software (StataCorp, College Station, TX, USA). 

### 2.4. Ethics Approval 

The study was conducted in accordance with the Declaration of Helsinki and approved by the Institutional Review Board of Nanyang Technological University Singapore, Singapore (IRB-2015-11-028). Written informed consent was obtained from all study participants prior to the commencement of data collection.

## 3. Results

### 3.1. Characteristics of Study Participants

We studied 329 working adults in Singapore. The sociodemographic characteristics of the study population are shown in Table 1. The age of the sample ranged between 22 and 65 years with a mean age of 40.7 (SD:11.1) years, and 78.1% were male. Reflecting national representation, a large proportion were Chinese (65.7%), followed by Malays (18.2%), Indians (11.9%), and other Asian groups (4.3%). The majority of participants were married (64.7%), had at least postsecondary education (89.7%), and were earning a monthly income < S$4,000 (69.0%). 

The overall PCS and MCS scores for the sample were 51.3 (SD: 6.2) and 50.2 (SD: 7.5), respectively. Table 2 shows the objectively and subjectively measured sleep parameters of the study population. According to study participants objective sleep measurements, participants slept an average of 5.8 (SD: 0.9) hours per night, were awake after sleep onset for an average of 37.9 (IQR: 28.1–49.5) minutes per night, and had a sleep efficiency of 79.1% (SD: 7.5). The mean PSQI global score was 5.1 (SD: 2.8). The results of the univariate analysis for PCS and MCS are provided in the Supplementary Materials (See Table S2).

### 3.2. Relationship between Objective and Subjective Measurements of Sleep

Subjective and objective sleep measurements were either weakly or not significantly correlated. There was a small but significant correlation between objectively and subjectively measured sleep duration (r = 0.27, *p* < 001), while there was no association between objectively and subjectively measured sleep efficiency. There was no association between objectively measured wake after sleep onset and subjectively assessed sleep disturbances.

### 3.3. Association between Sleep Parameters and Physical Health-Related Quality of Life

Table 3 shows the association between the various sleep parameters and physical health-related quality of life. In unadjusted models, higher scores (indicating a worse sleep quality), in five of the seven of the subjectively assessed domains of sleep (subjective sleep quality, sleep latency, sleep duration, sleep disturbances, and daytime dysfunction) were associated with impaired physical health-related quality of life. In fully adjusted models, subjectively assessed sleep disturbances were associated with impaired physical health-related quality of life, while higher, objectively measured, sleep efficiency was associated with greater physical health-related quality of life. The results of the multivariate analysis for objective and subjective sleep parameters and physical health-related quality of life are provided in the Supplementary Materials (See Tables S3 and S4).

### 3.4. Association between Sleep Parameters and Mental Health-Related Quality of Life

Table 4 shows the association between the various sleep parameters and mental health-related quality of life. In unadjusted models, higher scores (indicating a worse sleep quality), in five of the seven of the subjectively assessed domains of sleep (subjective sleep quality, sleep latency, sleep duration, sleep disturbances, and daytime dysfunction) were associated with impaired mental health-related quality of life. In fully adjusted models, a higher score for subjectively assessed daytime dysfunction was associated with impaired mental health-related quality of life. The results of the multivariate analysis for objective and subjective sleep parameters and mental health-related quality of life are provided in the Supplementary Materials (See Tables S5 and S6).

## 4. Discussion

### 4.1. Main Findings

The present study studied a non-clinical sample of 329 fulltime workers in Singapore to explore if objectively and subjectively measured sleep parameters were associated with physical and mental health-related quality of life. The findings confirmed that subjectively reported sleep disturbances were associated with a lower physical health-related quality of life. The findings also confirmed that higher sleep efficiency, objectively measured, was associated with greater physical health-related quality of life. The PSQI domain daytime dysfunction was associated with a lower mental health-related quality of life. The mean (SD) PSQI score in this study was 5.1 (SD: 2.8), which is in line with scores reported in the general population in Singapore [39], and significantly lower than what is observed from clinical populations and studies with older adults in Singapore [40,41].

### 4.2. Evidence on the Impacts of Sleep in Physical and Mental Health-Related Quality of Life

In our sample, we found a statistically significant association between sleep disturbances and physical health-related quality of life. This is not surprising as previous research has reported that physical health-related quality of life declines as sleep disturbances and insomnia become chronic [21,24]. The subscale sleep disturbances is quite broad in its composition and comprises many aspects of sleep quality. Each question that is used in the computation of the sleep disturbances subscale is aimed at eliciting possible underlying medical and physical conditions, sleep disorders, psychological or behavioural issues, environmental factors or a combination of any of these aspects, which can impact sleep adversely. Although a number of questions contributing towards the sleep disturbances subscale focuses on health problems that impact subjective sleep perception, it might be the case that the association of this subscale with physical health-related quality of life is mediated by underlying health conditions. Mental health-related quality of life is also reported to be impacted by sleep disturbances and insomnia, although only at the onset of sleep disturbances and insomnia with mental health-related quality of life stabilizing subsequently [24]. We also observed a significant association between sleep efficiency and physical health-related quality of life. Lower sleep efficiency indicates a higher sleep onset latency and more time awake after sleep onset, both of which are associated with anxiety-related insomnia, and lower sleep efficiency is also associated with sleep fragmentation clinical disorders, the most common being obstructive sleep apnea [42]. Lower sleep efficiency has also been found to be associated with metabolic syndrome [43] and all-cause mortality [44]. As reported in epidemiological literature, the effects of sleep not only impact our physical, but also our mental health [17,25,45]. The significant association between daytime dysfunction and mental health-related quality of life is not surprising as the daytime dysfunction subscale comprises of one question regarding fatigue and motivation and another question about trouble staying awake and daytime sleepiness, both of which may indicate depressive symptomatology. It is also well documented that individuals experiencing daytime dysfunction as a result of sleepiness are more likely to present depressive symptoms [18,22,33,46]. One cause of daytime dysfunction is insufficient sleep, and participants in our study averaged 5.8 h of sleep a night, more than an hour short of the 7 to 9 h recommended for optimal health [47]. It should also be acknowledged that there is sufficient evidence to suggest a relationship of bidirectionality in the association of impaired sleep and poor physical and mental health-related quality of life [48,49,50,51].

### 4.3. The Application of Complimentary Sleep Measurements

Our approach to examining sleep and health-related quality of life is novel in terms of applying both subjective and objective measurements of sleep to examine their relationship. Prior research has highlighted discrepancies between objective and subjective sleep measurements [28,52,53], and the use of both methods provides additional dimensions from which to approach and examine the relationship between sleep and health-related quality of life. Strengths and weaknesses are present in both objective and subjective measurements of sleep, sleep quantity determined by self-report questionnaires is unable to differentiate between time spent awake in bed and time asleep, whereas actigraphy data is unable to accurately identify if an individual suffers from sleep disturbances. The coupling of both methods captures different aspects of sleep and complement each other to provide a more complete and holistic picture of an individual’s sleep. Furthermore, their combined use will very likely raise the chances of identifying people with problematic sleep practices or sleep disorders.

The application of subjective and objective sleep measurements in parallel to each other in this study identified associations between domains of health-related quality of life and subjectively measured sleep disturbances and daytime dysfunction, and objectively measured sleep efficiency. Higher objective, but not subjective, sleep efficiency was associated with greater physical health-related quality of life. This demonstrates the added benefit of a multifactorial approach to measuring sleep, as the use of a subjective measurement alone did not show a relationship with physical health-related quality of life. While subjective and objective sleep measurements share commonalities and are conceptually equivalent, previous research has demonstrated inconsistencies between the measurements [15,28,52,53,54,55]. In line with previous research, our study found that subjective and objective sleep measurements were either weakly or not significantly correlated [15,28,52,53,54,55]. Previous studies examining the agreement between objective and subjective sleep measurements have reported both under- and overestimations of sleep efficiency [28,52]. In a study of older adults, 54% of participants underestimated their sleep efficiency by 5% or more as compared with what was objectively observed by actigraphy, whereas 27% of participants overestimated their sleep efficiency by 5% or more [28]. Previous research has shown that individuals with insomnia tend to underestimate their sleep duration and overestimate their sleep latency [54] which results in an overall underestimation of sleep efficiency. The under and overestimating of various aspects of sleep highlight the need for objective sleep measurements to supplement subjective measurements.

### 4.4. Strengths and Weaknesses

The present study has some notable strengths. The study included a multifactorial approach to sleep, encompassing both subjective and objective sleep parameters. Secondly, the study involved working individuals rather than individuals from disease cohorts. Thus, the results from this study may be more generalizable to the general population than those arising from studies of specific clinical populations. Finally, to our knowledge, this is the first study that studied the effects of both objective and subjective sleep parameters and health-related quality of life in a working population in Singapore. This study, however, is not without its limitations. Firstly, although the study used standardised and validated questionnaires, self-report questionnaires are susceptible to recall and social desirability bias. Secondly, one-time reporting of sleep variables may not accurately reflect the dynamic changes in sleep pattern, and thus long term effects of quality and quantity of sleep on physical and mental health-related quality of life, in addition to not being able to establish temporality and causality. Thirdly, the PSQI elicits responses from participants that best reflect their sleep during the previous month. The eight-day period of actigraphic recording was, thus, not aligned over the same reporting period of the PSQI. Furthermore, current recommendations for improved actigraphic assessment of sleep quality call for 14-day recordings to account for day-to-day, as well as week-to-week, variability in sleep quality [56]. Additionally, almost 80% of study participants were men, as the industries are comprised mainly of positions taken up by men such as engineers, technicians, and traffic controllers. Therefore, results may not be as generalizable to the female population of workers in Singapore. Lastly, the SD scores of the mean PCS and MCS scores were quiet small and this may be the result of using 2009 U.S. norms for scoring PCS and MCS. Ideally PCS and MCS scoring should be generated based on norms from the local population, however, this option was unavailable to us at the time of the study.

### 4.5. Future Directions

For future research, it would be necessary to replicate this study with a larger sample, with workers from varying industries, and applying recruitment strategies to ensure a greater percentage of females are enrolled. As this study only included a single self-report instrument to assess sleep quality, additional self-report tools examining other aspects of sleep or specific disorders, for example, sleep apnea or insomnia, should be considered in future studies of the relationship between sleep and health-related quality of life. Sleep hygiene education in workplaces in Singapore has the potential to improve both the quality and quantity of worker's sleep and may also help improve worker’s health-related quality of life, and therefore the effects should be studied in a longitudinal fashion. Furthermore, in a longitudinal design, the bidirectional relationship between sleep and health-related quality of life may be better understood.

Further study is needed to examine the workplace factors which may be contributing to sleep disturbances, the mechanistic pathways linking sleep disturbances and reduced health-related quality of life, as well as the impact of low health-related quality of life on work performance and productivity. These will better inform the allocation of resources for an increased outreach of educational efforts aimed at improving sleep health literacy and modifying workplace health policies to improve sleep health and quality of life among workers in Singapore.

## 5. Conclusions

The application of subjective and objective sleep measurements in parallel identified a number of sleep parameters that were associated with health-related quality of life, even after adjusting for numerous confounders related to sociodemographic, health, lifestyle, and occupational factors. Subjectively reported sleep disturbances were associated with a lower physical health-related quality of life, whereas higher objective, but not subjective, sleep efficiency was associated with greater physical health-related quality of life. The PSQI domain “daytime dysfunction” was independently associated with a lower mental health-related quality of life. More research is necessary to explore the mechanistic pathways linking sleep impairment and reduced health-related quality of life, as well as the impact of health-related quality of life on work performance and productivity. Workplace health promotion planners should consider programmes that educate workers on better sleep hygiene practices in an effort to improve sleep quality and health-related quality of life.

## Figures and Tables

**Table 1:** Characteristics of study participants and physical component summary (PCS) and mental component summary (MCS) scores.

Demographics	Total	PCS (Mean ± SD)	MCS (Mean ± SD)
Age (years)	40.7 ± 11.1		
21–30	84 (28.0)	52.1 ± 5.5	48.8 ± 7.2
31–40	98 (28.9)	50.8 ± 6.9	49.1 ± 7.0
40+	147 (43.2)	51.2 ± 6.1	51.6 ± 7.9
Gender			
Male	256 (77.8)	51.3 ± 6.4	49.7 ± 7.6
Female	73 (22.2)	51.5 ± 5.5	51.7 ± 7.1
Education			
Primary and secondary	32 (10.3)	52.0 ± 5.0	51.4 ± 9.1
Pre-college	178 (54.1)	50.2 ± 6.4	50.3 ± 7.6
College and above	119 (35.6)	52.9 ± 5.8	49.5 ± 7.0
Ethnicity			
Chinese	216 (65.7)	52.0 ± 5.9	50.2 ± 7.7
Malay	60 (18.2)	49.3 ± 6.7	49.9 ± 8.0
Indian	39 (11.9)	50.1 ± 6.8	49.4 ± 5.7
Others ^a^	14 (4.3)	53.0 ± 5.1	52.5 ± 9.0
Marital status			
Single ^b^	116 (35.3)	52.3 ± 5.8	50.2 ± 7.3
Married	213 (64.7)	50.8 ± 6.3	50.1 ± 7.7
Monthly income			
<S$4,000	227 (69.0)	51.2 ± 6.0	49.9 ± 7.9
≥S$4,000	102 (31.0)	51.7 ± 6.5	50.7 ± 6.9

Continuous variables are presented as mean ± standard deviation, and categorical variables as n (%); ^a^ includes mixed ethnicities, Indonesians, Pakistanis and Filipinos; ^b^ includes never married, widowed, and divorced; SD, standard deviation.

**Table 2:** Distribution of objective and subjective sleep parameters

Sleep Parameters	Total
Objective sleep parameters	
Total sleep time (hours), mean (SD)	5.8 ± 0.9
<5 h	53 (19.0)
5–6 h	111 (40.0)
6–7 h	97 (34.8)
>7 h	18 (6.5)
Wake after sleep onset (min), median (IQR)	37.9 (28.1–49.5)
Sleep efficiency (%)	80.9 ± 7.5
Subjective sleep parameters	
PSQI global score	5.1 ± 2.8
Subjective sleep quality, median (IQR)	1 (0–2)
Sleep latency, median (IQR)	1 (0–1)
Sleep duration, median (IQR)	1 (0–2)
Habitual sleep efficiency, median (IQR)	0 (0–1)
Sleep disturbances, median (IQR)	1 (1–1)
Use of sleeping medications, median (IQR)	0 (0–0)
Daytime dysfunction, median (IQR)	1 (0–1)

IQR, inter-quartile range; SD, standard deviation.

**Table 3:** Association between objective and subjective sleep parameters on physical health-related quality of life.

Sleep Parameters	Standardized β (95% CI)	Standardized β_adj_ (95% CI) ^
Subjective sleep parameters		
Subjective sleep quality	−2.720 (−3.835, −1.605) ***	−1.416 (−2.846, 0.014)
Sleep latency	−1.771 (−2.518, −1.024) ***	−0.704 (−1.522, 0.114)
Sleep duration	−1.364 (−2.067, −0.661) ***	−0.416 (−1.220, 0.387)
Habitual sleep efficiency	−0.072 (−0.912, 0.768)	0.697 (−0.118, 1.512)
Sleep disturbances	−3.430 (−4.598, −2.262) ***	−1.829 (−3.216, −0.443) *
Use of sleeping medications	−1.294 (−2.883, 0.295)	−0.593 (−2.052, 0.866)
Daytime dysfunction	−1.861 (−2.835, −0.886) ***	−0.909 (−1.948, 0.130)
Objective sleep parameters		
Total sleep time (hours)	0.266 (−0.559, 1.090)	0.777 (−1.802, 0.248)
Wake after sleep onset (min)	0.002 (−0.041, 0.045)	0.049 (−0.007, 0.106)
Sleep efficiency (%)	0.125 (0.030, 0.220) *	0.211 (0.050, 0.372) *

^ Adjusted for sociodemographic, health, lifestyle, and occupational factors. * *p* < 0.05. *** *p* < 0.001.

**Table 4:** Association between objective and subjective sleep parameters on mental health-related quality of life.

Sleep Parameters	Standardized β (95% CI) ^	Standardized β_adj_ (95% CI) ^
Subjective sleep parameters		
Subjective sleep quality	−4.726 (−6.037, –3.415) ***	−1.415 (−2.939, 0.110)
Sleep latency	−2.203 (−3.114, −1.293) ***	−0.809 (−1.704, 0.086)
Sleep duration	−1.282 (−2.148, −0.416) ***	−0.027 (−0.891, 0.838)
Habitual sleep efficiency	−0.224 (−1.249, 0.800)	0.526 (−0.341, 1.394)
Sleep disturbances	−2.695 (−4.163, −1.228) ***	0.243 (−1.168, 1.653)
Use of sleeping medications	−1.434 (−3.375, 0.506)	−0.722 (−2.221, 0.776)
Daytime dysfunction	−5.243 (−6.316, −4.170) ***	−2.945 (−4.136, −1.754) ***
Objective sleep parameters		
Total sleep time (hours)	−0.374 (−1.373, 0.626)	−0.140 (−1.376, 1.096)
Wake after sleep onset (mins)	0.020 (−0.032, 0.071)	0.052 (−0.005, 0.110)
Sleep efficiency (%)	0.019 (−0.097, 0.136)	0.023 (−0.131, 0.177)

^ Adjusted for sociodemographic, health, lifestyle, and occupational factors. *** *p* < 0.001.

## Supplementary Materials

The following are available online at https://www.mdpi.com/1660-4601/16/21/4147/s1, Table S1: Characteristics of study participants (Health, lifestyle and occupational factors), Table S2: Univariate associations with Physical Component Summary (PCS) and Mental Component Summary (MCS), Table S3: Multivariable analysis for objective sleep parameters and Physical Component Summary, Table S4: Multivariable analysis for subjective sleep parameters and Physical Component Summary, Table S5: Multivariable analysis for objective sleep parameters and Mental Component Summary, Table S6: Multivariable analysis for subjective sleep parameters and Mental Component Summary.
