# Supplementary material for: A Multifactorial Approach to Sleep and Its Association with Health-Related Quality of Life in a Multiethnic Asian Working Population: A Cross-Sectional Analysis
Source: Int J Environ Res Public Health. 2019 Oct 28;16(21):4147. doi: 10.3390/ijerph16214147 (PMC6862149; doi:10.3390/ijerph16214147)
Supplement: Supplementary file 1 [file ijerph-16-04147-s001.pdf]

Table S1: Characteristics of study participants (Health, lifestyle and occupational factors)

	Total
**Health and lifestyle factors**	
Smoking	
Non/ex-smoker	222 (67.5)
Current smoker	107 (32.5)
Alcohol drinking	
Non-drinker	169 (50.6)
Drinks less than once a month	107 (32.0)
Drinks once or more a month	58 (17.4)
Physical activity[a]	
Low	107 (32.6)
Moderate	129 (39.3)
High	92 (28.1)
Self-reported comorbidities	
0	200 (60.8)
≥1	129 (39.2)
Overweight and obese	
No (BMI <23.0)	104 (31.3)
Yes (BMI ≥23.0)	228 (68.7)
Stress at home in previous 12 months	
Never	120 (36.5)
Some	188 (57.1)
Several periods	21 (6.4)
Current level of financial stress	
None	107 (32.5)
Some	140 (42.6)
Moderate or severe	82 (24.9)
**Occupational factors**	
Shift work	
No	227 (68.0)
Yes	107 (32.0)
Job type	
Office	160 (47.9)
Control room	100 (29.9)
Workshop	74 (22.2)
Stress at work in previous 12 months	
None	67 (20.4)
Some	210 (63.8)
Several periods	52 (15.8)
Years employed in the current company, median (IQR)	4.9 (2.3-9.4)
Daily working hours	8.6 ± 1.4
Work location	
Aboveground	228 (68.3)
Underground	106 (31.7)

Values are presented as mean ± standard deviation (normally distributed) or median [inter-quartile range] (skewed) for continuous variables, and number (%) for categorical variables.
BMI: body mass index
[a]Physical activity: Low=Metabolic equivalent task mins per week <600, Moderate=Metabolic equivalent task mins per week 600-2999, High=Metabolic equivalent task mins per week ≥3000.

Table S2: Univariate associations with Physical Component Summary (PCS) and Mental Component Summary (MCS)

	Dependent Variable: PCS			Dependent Variable: MCS		
Variable	Beta	Std. Err.	P[a]	Beta	Std. Err.	P[a]
**Socio-demographic factors**						
Age (years)						
21-30	Ref	Ref	Ref	Ref	Ref	Ref
31-40	-2.228	0.920	0.182	0.299	1.045	0.775
≥41	-0.792	0.784	0.313	2.872	1.001	0.004**
Gender						
Male	Ref	Ref	Ref	Ref	Ref	Ref
Female	0.169	0.822	0.837	1.989	0.992	0.046*
Education						
Primary and secondary	Ref	Ref	Ref	Ref	Ref	Ref
Pre-college	-1.768	1.150	0.125	-1.242	1.426	0.385
College and above	1.039	1.190	0.383	-1.953	1.475	0.186
Ethnicity						
Chinese	Ref	Ref	Ref	Ref	Ref	Ref
Malay	-2.880	0.884	0.001**	-0.456	1.092	0.676
Indian	-1.810	1.049	0.085	-0.766	1.296	0.555
Others[b]	0.978	1.681	0.561	2.285	2.076	0.272
Marital status						
Single[c]	Ref	Ref	Ref	Ref	Ref	Ref
Married	-1.304	0.710	0.067	-0.095	0.867	0.913
Monthly income						
<S$4,000	Ref	Ref	Ref	Ref	Ref	Ref
≥S$4,000	0.395	0.732	0.590	0.803	0.888	0.367
**Health and lifestyle factors**						
Smoking						
Non/ex-smoker	Ref	Ref	Ref	Ref	Ref	Ref
Current smoker	-1.551	0.730	0.034*	-0.680	0.896	0.448
Alcohol consumption						
Non-drinker	Ref	Ref	Ref	Ref	Ref	Ref
Drinks less than once a month	0.108	0.770	0.889	-1.180	0.932	0.206
Drinks once or more a month	-0.294	0.951	0.757	0.546	1.151	0.636
Physical activity[d]						
Low	Ref	Ref	Ref	Ref	Ref	Ref
Moderate	1.874	0.810	0.021*	1.275	0.992	0.200
High	0.620	0.881	0.482	0.799	1.080	0.460
Self-reported comorbidities						
0	Ref	Ref	Ref	Ref	Ref	Ref
≥1	-1.428	0.699	0.042*	-0.337	0.858	0.695
BMI	-0.170	0.063	0.007**	0.107	0.077	0.164

Stress at home in previous 12 months						
None	Ref	Ref	Ref	Ref	Ref	Ref
Some	-1.418	0.713	0.047*	-5.536	0.796	<0.001***
Several periods	-5.238	1.470	<0.001***	-12.179	1.640	<0.001***
Current level of financial stress						
None	Ref	Ref	Ref	Ref	Ref	Ref
Some	-2.151	0.781	0.006**	-3.862	0.898	<0.001***
Moderate or severe	-3.327	0.897	<0.001***	-7.798	1.032	<0.001***
**Occupational factors**						
Shift work						
No	Ref	Ref	Ref	Ref	Ref	Ref
Yes	-0.544	0.730	0.456	0.634	0.886	0.475
Job type						
Office	Ref	Ref	Ref	Ref	Ref	Ref
Control room	0.068	0.781	0.931	-0.045	0.964	0.963
Workshop	-2.634	0.861	0.002**	-0.799	1.062	0.452
Stress at work in previous 12 months						
None	Ref	Ref	Ref	Ref	Ref	Ref
Some	-0.676	0.871	0.438	-4.311	0.988	<0.001***
Several periods	-1.123	1.159	0.333	-9.433	1.315	<0.001***
Years employed in the current company						
<3	Ref	Ref	Ref	Ref	Ref	Ref
≥3	-0.704	0.740	0.342	0.175	0.905	0.847
Daily working hours	-0.250	0.247	0.313	-0.422	0.306	0.168
Work location						
Aboveground	Ref	Ref	Ref	Ref	Ref	Ref
Underground	-0.941	0.728	0.197	-0.299	0.887	0.736

BMI: body mass index

[a] P was derived using Generalized Linear Modelling.

[b] Includes mixed ethnicities, Indonesians, Pakistanis and Filipinos

[c] Includes never married, widowed and divorced

[d] Physical activity: Low=Metabolic equivalent task mins per week <600, Moderate=Metabolic equivalent task mins per week 600-2999, High=Metabolic equivalent task mins per week ≥3000.

*p<0.05. **p<0.01. ***p<0.001.

Table S3: Multivariable analysis for objective sleep parameters and Physical Component Summary

Variable	Model 1 Beta (p[a])	Model 2 Beta (p[a])	Model 3 Beta (p[a])	Model 4 Beta (p[a])
**Socio-demographic factors**				
Age (years)				
21-30	1.00	1.00	1.00	1.00
31-40	-1.034 (0.294)	-0.761 (0.441)	-0.698 (0.480)	-0.055 (0.960)
≥41	-0.290 (0.786)	-0.136 (0.906)	-0.163 (0.886)	-0.036 (0.978)
Gender				
Male	1.00	1.00	1.00	1.00
Female	-0.758 (0.319)	-1.221 (0.135)	-2.175 (0.012)*	-1.488 (0.092)
Education				
Primary and secondary	1.00	1.00	1.00	1.00
Pre-college	-1.464 (0.188)	-1.409 (0.229)	-2.226 (0.063)	-2.094 (0.087)
College and above	-0.701 (0.585)	-0.576 (0.673)	-0.700 (0.618)	-0.486 (0.742)
Ethnicity				
Chinese	1.00	1.00	1.00	1.00
Malay	-2.263 (0.036)*	-1.943 (0.106)	-1.423 (0.235)	0.038 (0.979)
Indian	-1.316 (0.248)	-1.226 (0.263)	-0.373 (0.731)	0.271 (0.808)
Others[b]	0.952 (0.500)	1.661 (0.235)	2.429 (0.077)	3.064 (0.037)*
Marital status				
Single[c]	1.00	1.00	1.00	1.00
Married	-0.690 (0.395)	-0.850 (0.301)	-0.707 (0.402)	-0.981 (0.261)
Monthly income				
<S$4,000	1.00	1.00	1.00	1.00
≥S$4,000	-0.414 (0.622)	-0.057 (0.948)	-0.415 (0.645)	-0.587 (0.520)
**Health and lifestyle factors**				
Smoking				
Non/ex-smoker	--	1.00	--	--
Current smoker		-0.017 (0.984)		
Alcohol drinking				
Non-drinker	--	1.00	1.00	--
Drinks less than once a month		-0.720 (0.365)	-0.823 (0.290)	
Drinks once or more a month		-1.231 (0.231)	-1.082 (0.276)	
Physical activity[d]				
Low	--	1.00	1.00	1.00
Moderate		1.448 (0.065)	1.363 (0.082)	1.735 (0.042)*
High		0.897 (0.306)	1.123 (0.223)	0.454 (0.649)
Self-reported comorbidities				
0	--	1.00	1.00	1.00
≥1		-1.305 (0.069)	-1.251 (0.077)	-1.589 (0.042)*
BMI	--	-0.100 (0.207)	-0.113 (0.145)	-0.019 (0.807)
Stress at home in previous 12 months				
None		1.00	1.00	1.00
Some	--	-0.727 (0.324)	-0.396 (0.608)	-0.553 (0.490)
Several periods		-3.687 (0.053)	-3.318 (0.075)	-3.252 (0.157)
Current level of financial stress				
None	--	1.00	1.00	

Some		-1.784 (0.035)*	-1.925 (0.027)*	-2.085 (0.026)*
Moderate or severe		-1.675 (0.082)	-1.604 (0.126)	-1.535 (0.145)
**Occupational factors**				
Shift work				
No	--	--	1.00	--
Yes			-612 (0.600)	
Job type				
Office	--	--	1.00	1.00
Control room			0.500 (0.682)	-0.546 (0.537)
Workshop			-2.806 (0.015)*	-3.058 (0.009)**
Stress at work in previous 12 months				
None			1.00	--
Some			-0.334 (0.732)	
Several periods			0.082 (0.954)	
Years employed in the current company				
<3	--	--	1.00	--
≥3			-0.370 (0.655)	
Daily working hours	--	--	-0.595 (0.039)*	-0.489 (0.154)
Work location				
Aboveground	--	--	1.00	--
Underground			-0.776 (0.293)	
**Sleep parameters**				
Total sleep time (hours)	--	--	--	-0.777 (0.137)
Wake after sleep onset (mins)	--	--	--	0.049 (0.086)
Sleep efficiency (%)	--	--	--	0.211 (0.010)*

BMI: body mass index

[a] P was derived using Generalized Linear Modelling.

[a] Includes mixed ethnicities, Indonesians, Pakistanis and Filipinos

[b] Includes never married, widowed and divorced

[c] Physical activity: Low=Metabolic equivalent task mins per week <600, Moderate=Metabolic equivalent task mins per week 600-2999, High=Metabolic equivalent task mins per week ≥3000.

*p<0.05. **p<0.01. ***p<0.001.

Table S4: Multivariable analysis for subjective sleep parameters and Physical Component Summary

Variable	Model 1 Beta (p[a])	Model 2 Beta (p[a])	Model 3 Beta (p[a])	Model 4 Beta (p[a])
**Socio-demographic factors**				
Age				
21-30	1.00	1.00	1.00	1.00
31-40	-1.034 (0.294)	-0.761 (0.441)	-0.698 (0.480)	-0.243 (0.795)
≥41	-0.290 (0.786)	-0.136 (0.906)	-0.163 (0.886)	0.147 (0.891)
Gender				
Male	1.00	1.00	1.00	1.00
Female	-0.758 (0.319)	-1.221 (0.135)	-2.175 (0.012)*	-1.626 (0.044)*
Education				
Primary and secondary	1.00	1.00	1.00	1.00
Pre-college	-1.464 (0.188)	-1.409 (0.229)	-2.226 (0.063)	-1.646 (0.160)
College and above	-0.701 (0.585)	-0.576 (0.673)	-0.700 (0.618)	0.219 (0.870)
Ethnicity				
Chinese	1.00	1.00	1.00	1.00
Malay	-2.263 (0.036)*	-1.943 (0.106)	-1.423 (0.235)	-1.252 (0.302)
Indian	-1.316 (0.248)	-1.226 (0.263)	-0.373 (0.731)	-1.253 (0.250)
Others[b]	0.952 (0.500)	1.661 (0.235)	2.429 (0.077)	2.177 (0.100)
Marital status				
Single[c]	1.00	1.00	1.00	1.00
Married	-0.690 (0.395)	-0.850 (0.301)	-0.707 (0.402)	-0.822 (0.263)
Monthly income				
<S$4,000	1.00	1.00	1.00	1.00
≥S$4,000	-0.414 (0.622)	-0.057 (0.948)	-0.415 (0.645)	-0.772 (0.335)
**Health and lifestyle factors**				
Smoking				
Non/ex-smoker	--	1.00	--	--
Current smoker		-0.017 (0.984)		
Alcohol drinking				
Non-drinker	--	1.00	1.00	--
Drinks less than once a month		-0.720 (0.365)	-0.823 (0.290)	
Drinks once or more a month		-1.231 (0.231)	-1.082 (0.276)	
Physical activity[d]				
Low	--	1.00	1.00	1.00
Moderate		1.448 (0.065)	1.363 (0.082)	1.074 (0.155)
High		0.897 (0.306)	1.123 (0.223)	1.152 (0.180)
Self-reported comorbidities				
0	--	1.00	1.00	1.00
≥1		-1.305 (0.069)	-1.251 (0.077)	-0.851 (0.180)
BMI	--	-0.100 (0.207)	-0.113 (0.145)	-0.079 (0.273)
Stress at home in previous 12 months				
None		1.00	1.00	1.00
Some	--	-0.727 (0.324)	-0.396 (0.608)	0.913 (0.220)
Several periods		-3.687 (0.053)	-3.318 (0.075)	-1.435 (0.398)
Current level of financial stress				
None	--	1.00	1.00	1.00

Some		-1.784 (0.035)*	-1.925 (0.027)*	-0.829 (0.329)
Moderate or severe		-1.675 (0.082)	-1.604 (0.126)	-0.248 (0.794)
**Occupational factors**				
Shift work				
No	--	--	1.00	--
Yes			-612 (0.600)	
Job type				
Office	--	--	1.00	1.00
Control room			0.500 (0.682)	0.442 (0.572)
Workshop			-2.806 (0.015)*	-2.272 (0.023)*
Stress at work in previous 12 months				
None			1.00	--
Some			-0.334 (0.732)	
Several periods			0.082 (0.954)	
Years employed in the current company				
<3	--	--	1.00	--
≥3			-0.370 (0.655)	
Daily working hours	--	--	-0.595 (0.039)*	-0.452 (0.101)
Work location				
Aboveground	--	--	1.00	--
Underground			-0.776 (0.293)	
**Sleep quality**				
Subjective sleep quality	--	--	--	-1.416 (0.052)
Sleep latency	--	--	--	-0.704 (0.092)
Sleep duration	--	--	--	-0.416 (0.310)
Habitual sleep efficiency	--	--	--	0.697 (0.094)
Sleep disturbances	--	--	--	-1.829 (0.010)*
Use of sleeping medications	--	--	--	-0.593 (0.426)
Daytime dysfunction	--	--	--	-0.909 (0.086)

BMI: body mass index

[a] P was derived using Generalized Linear Modelling.

[b] Includes mixed ethnicities, Indonesians, Pakistanis and Filipinos

[c] Includes never married, widowed and divorced

[d] Physical activity: Low=Metabolic equivalent task mins per week <600, Moderate=Metabolic equivalent task mins per week 600-2999, High=Metabolic equivalent task mins per week ≥3000.

*p<0.05. **p<0.01. ***p<0.001.

Table S5: Multivariable analysis for objective sleep parameters and Mental Component Summary

Variable	Model 1 Beta (p[a])	Model 2 Beta (p[a])	Model 3 Beta (p[a])	Model 4 Beta (p[a])
**Socio-demographic factors**				
Age				
21-30	1.00	1.00	1.00	1.00
31-40	1.220 (0.302)	1.213 (0.274)	1.195 (0.290)	1.187 (0.338)
≥41	3.723 (0.003)**	3.551 (0.002) **	3.449 (0.002) **	4.050 (0.001)
Gender				
Male	1.00	1.00	1.00	1.00
Female	1.942 (0.040) *	2.158 (0.019) *	2.339 (0.019)*	2.003 (0.055)
Education				
Primary and secondary	1.00	1.00	1.00	1.00
Pre-college	0.333 (0.848)	1.101 (0.472)	0.535 (0.711)	0.627 (0.670)
College and above	-0.237 (0.899)	0.677 (0.697)	0.449(0.786)	1.152 (0.509)
Ethnicity				
Chinese	1.00	1.00	1.00	1.00
Malay	0.733 (0.570)	1.614 (0.207)	1.569 (0.190)	1.171(0.393)
Indian	-0.132 (0.909)	-0.340 (0.743)	-0.461 (0.673)	-1.214 (0.308)
Others[b]	3.111 (0.209)	3.162 (0.107)	3.585 (0.039)*	2.529 (0.174)
Marital status				
Single[c]	1.00	1.00	1.00	1.00
Married	-1.757 (0.074)	-0.577 (0.542)	-0.571 (0.544)	-0.749 (0.461)
Monthly income				
<S$4,000	1.00	1.00	1.00	1.00
≥S$4,000	0.731 (0.407)	0.420 (0.623)	1.476 (0.105)	0.997 (0.316)
**Health and lifestyle factors**				
Smoking				
Non/ex-smoker	--	1.00	--	--
Current smoker		0.380 (0.673)		
Alcohol drinking				
Non-drinker	--	1.00	1.00	1.00
Drinks less than once a month		-0.338 (0.700)	-0.441 (0.614)	-0.576 (0.534)
Drinks once or more a month		1.354 (0.223)	1.267 (0.234)	1.256 (0.307)
Physical activity[d]				
Low	--	1.00	--	--
Moderate		0.668 (0.419)		
High		0.531 (0.616)		
Self-reported comorbidities				
0	--	1.00	--	--
≥1		-0.445 (0.587)		
BMI	--	0.043 (0.550)	--	--
Stress at home in previous 12 months				
None		1.00	1.00	1.00
Some	--	-4.153 (<0.001)***	-3.855 (<0.001)***	-3.638 (<0.001)***
Several periods		-10.026 (<0.001)***	-8.959 (<0.001)***	-9.123 (<0.001)***
Current level of financial stress				
None	--	1.00	1.00	1.00
Some		-1.604 (0.059)	-1.365 (0.104)	-1.117 (0.233)

Moderate or severe		-4.598 (<0.001)***	-4.116 (<0.001)***	-4.742 (<0.001)***
**Occupational factors**				
Shift work				
No	--	--	1.00	1.00
Yes			1.483 (0.180)	0.991 (0.285)
Job type				
Office	--	--	1.00	--
Control room			-1.155 (0.337)	
Workshop			-1.040 (0.370)	
Stress at work in previous 12 months				
None			1.00	1.00
Some			-1.251 (0.232)	-1.679 (0.148)
Several periods			-4.905 (0.002) **	-3.780 (0.037)*
Years employed in the current company				
<3	--	--	1.00	1.00
≥3			-1.532 (0.095)	-1.680 (0.092)
Daily working hours	--	--	-0.706 (0.007)**	-0.640 (0.012)*
Work location				
Aboveground	--	--	1.00	1.00
Underground			1.098 (0.206)	1.070 (0.246)
**Sleep parameters**				
Total sleep time (hours)	--	--	--	-0.140 (0.824)
Wake after sleep onset (mins)	--	--	--	0.052 (0.086)
Sleep efficiency (%)	--	--	--	0.023 (0.771)

BMI: body mass index

[a] P was derived using Generalized Linear Modelling.

[b] Includes mixed ethnicities, Indonesians, Pakistanis and Filipinos

[c] Includes never married, widowed and divorced

[d] Physical activity: Low=Metabolic equivalent task mins per week <600, Moderate=Metabolic equivalent task mins per week 600-2999, High=Metabolic equivalent task mins per week ≥3000.

*p<0.05. **p<0.01. ***p<0.001.

Table S6: Multivariable analysis for subjective sleep parameters and Mental Component Summary

Variable	Model 1<br>Beta (p[a])	Model 2<br>Beta (p[a])	Model 3<br>Beta (p[a])	Model 4<br>Beta (p[a])
**Socio-demographic factors**				
Age				
21-30	1.00	1.00	1.00	1.00
31-40	1.220 (0.302)	1.213 (0.274)	1.195 (0.290)	1.159 (0.269)
≥41	3.723 (0.003)**	3.551 (0.002) **	3.449 (0.002) **	3.412 (0.002)**
Gender				
Male	1.00	1.00	1.00	1.00
Female	1.942 (0.040) *	2.158 (0.019) *	2.339 (0.019)*	2.611 (0.003)**
Education				
Primary and secondary	1.00	1.00	1.00	1.00
Pre-college	0.333 (0.848)	1.101 (0.472)	0.535 (0.711)	0.958 (0.504)
College and above	-0.237 (0.899)	0.677 (0.697)	0.449(0.786)	1.642 (0.315)
Ethnicity				
Chinese	1.00	1.00	1.00	1.00
Malay	0.733 (0.570)	1.614 (0.207)	1.569 (0.190)	1.101 (0.307)
Indian	-0.132 (0.909)	-0.340 (0.743)	-0.461 (0.673)	-1.512 (0.133)
Others[b]	3.111 (0.209)	3.162 (0.107)	3.585 (0.039)*	3.985 (0.026)*
Marital status				
Single[c]	1.00	1.00	1.00	1.00
Married	-1.757 (0.074)	-0.577 (0.542)	-0.571 (0.544)	-0.450 (0.605)
Monthly income				
<S$4,000	1.00	1.00	1.00	1.00
≥S$4,000	0.731 (0.407)	0.420 (0.623)	1.476 (0.105)	0.723 (0.399)
**Health and lifestyle factors**				
Smoking				
Non/ex-smoker	--	1.00	--	--
Current smoker		0.380 (0.673)		
Alcohol drinking				
Non-drinker	--	1.00	1.00	1.00
Drinks less than once a month		-0.338 (0.700)	-0.441 (0.614)	-0.229 (0.784)
Drinks once or more a month		1.354 (0.223)	1.267 (0.234)	1.279 (0.211)
Physical activity[d]				
Low	--	1.00	--	--
Moderate		0.668 (0.419)		
High		0.531 (0.616)		
Self-reported comorbidities				
0	--	1.00	--	--
≥1		-0.445 (0.587)		
BMI	--	0.043 (0.550)	--	--
Stress at home in previous 12 months				
None		1.00	1.00	1.00
Some	--	-4.153 (<0.001)***	-3.855 (<0.001)***	-2.610 (0.002)**
Several periods		-10.026 (<0.001)***	-8.959 (<0.001)***	-7.030 (0.001)**
Current level of financial stress				
None	--	1.00	1.00	1.00
Some		-1.604 (0.059)	-1.365 (0.104)	0.032 (0.970)

Moderate or severe		-4.598 (<0.001)***	-4.116 (<0.001)***	-2.457 (0.030)*
**Occupational factors**				
Shift work				
No	--	--	1.00	1.00
Yes			1.483 (0.180)	1.308 (0.075)
Job type				
Office	--	--	1.00	--
Control room			-1.155 (0.337)	
Workshop			-1.040 (0.370)	
Stress at work in previous 12 months				
None			1.00	1.00
Some			-1.251 (0.232)	-0.828 (0.380)
Several periods			-4.905 (0.002)**	-3.414 (0.018)*
Years employed in the current company				
<3	--	--	1.00	1.00
≥3			-1.532 (0.095)	-1.190 (0.155)
Daily working hours	--	--	-0.706 (0.007)**	-0.574 (0.017)*
Work location				
Aboveground	--	--	1.00	1.00
Underground			1.098 (0.206)	0.967 (0.235)
**Sleep quality**				
Subjective sleep quality	--	--	--	-1.415 (0.069)
Sleep latency	--	--	--	-0.809 (0.076)
Sleep duration	--	--	--	-0.027 (0.952)
Habitual sleep efficiency	--	--	--	0.526 (0.235)
Sleep disturbances	--	--	--	0.243 (0.736)
Use of sleeping medications	--	--	--	-0.722 (0.345)
Daytime dysfunction	--	--	--	-2.945 (<0.001)***

BMI: body mass index

[a] P was derived using Generalized Linear Modelling.

[b] Includes mixed ethnicities, Indonesians, Pakistanis and Filipinos

[c] Includes never married, widowed and divorced

[d] Physical activity: Low=Metabolic equivalent task mins per week <600, Moderate=Metabolic equivalent task mins per week 600-2999, High=Metabolic equivalent task mins per week ≥3000.

*p<0.05. **p<0.01. ***p<0.001.
